# Post-Transplant Vitamin D Deficiency in Lung Transplant Recipients: Impact on Outcomes and Prognosis

**DOI:** 10.3389/ti.2024.13313

**Published:** 2024-10-25

**Authors:** Min Seo Ki, Nam Eun Kim, Ala Woo, Song Yee Kim, Young Sam Kim, Ha Eun Kim, Jin Gu Lee, Hyo Chae Paik, Moo Suk Park

**Affiliations:** ^1^ Division of Pulmonary and Critical Care Medicine, Department of Internal Medicine, Severance Hospital, Yonsei University College of Medicine, Seoul, Republic of Korea; ^2^ Division of Pulmonology and Allergy, Department of Internal Medicine, National Health Insurance Service Ilsan Hospital, Goyang, Republic of Korea; ^3^ Division of Pulmonary and Critical Care Medicine, Department of Internal Medicine, Ewha Womans University College of Medicine, Ewha Womans Seoul Hospital, Seoul, Republic of Korea; ^4^ Department of Thoracic and Cardiovascular Surgery, Severance Hospital, Yonsei University College of Medicine, Seoul, Republic of Korea; ^5^ Department of Thoracic and Cardiovascular Surgery, Myongji Hospital, Goyang, Republic of Korea

**Keywords:** vitamin D deficiency, lung transplantation, survival, prognosis, pneumonia

## Abstract

Despite the recognized clinical significance of vitamin D deficiency in other solid organ transplant recipients, its specific relevance in lung transplantation remains to be fully understood. In this study, we performed a retrospective observational study on the impact of vitamin D deficiency on clinical outcomes and prognosis in 125 lung transplant recipients (LTRs) from October 2014 to March 2020 at a university hospital in Seoul, South Korea. Among 125 LTRs, 51 patients (40.8%) were vitamin D deficient. LTRs in the vitamin D-deficient group exhibited a higher incidence of post-transplant pneumonia and overall mortality than those with normal vitamin D levels during the follow-up period. This trend persisted when subjects were stratified into vitamin D tertiles. Furthermore, post-transplant vitamin D levels and C-reactive protein (CRP) significantly impacted pneumonia incidence and survival outcomes. Prognosis also varied based on cumulative vitamin D supplementation after transplantation, with patients receiving higher cumulative supplementation demonstrating improved prognosis. Our findings underscore the importance of assessing and maintaining optimal vitamin D levels post-transplantation, suggesting a potential avenue for improving outcomes in lung transplant recipients, especially in mitigating infection risk and enhancing long-term survival. Further research into optimal vitamin D levels and supplementation strategies in this population is warranted.

## Graphical Abstract

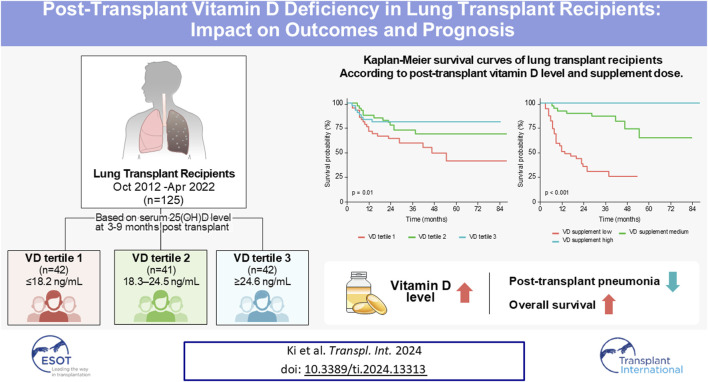

## Introduction

Beyond calcium homeostasis and bone metabolism, vitamin D deficiency is associated with numerous chronic diseases. Receptors and enzymes involved in vitamin D metabolism are broadly expressed in almost all tissues and cells *in vivo*, thus mediating various extraskeletal effects [1]. These include immunomodulatory and anti-infective properties, so vitamin D has been linked to major lung diseases and lung transplant status.

Vitamin D deficiency appears to be associated with the prognosis of various respiratory diseases, including chronic obstructive pulmonary disease (COPD) [2–4], bronchial asthma (BA) [5, 6], respiratory infections [7–9], and interstitial lung disease (ILD) [10, 11]. Vitamin D deficiency is frequently observed in solid organ transplant recipients as well. It has been reported to be related with an increased risk of acute rejection and infection, as well as overall survival in liver, kidney, and lung transplant recipients (LTRs) [12–14]. Studies have shown that solid organ transplant recipients who received vitamin D supplementation had a lower incidence of rejection than those who did not [12, 15].

However, high-dose vitamin D supplementation showed no significant difference from the placebo control group in chronic rejection and overall survival in a randomized controlled trial for LTRs [16]. It is unclear whether low vitamin D status in LTRs merely reflects the patient’s severity and poor health condition or is a risk factor independent of morbidity and mortality.

In this context, this study was performed with the aim of elucidating the clinical relevance of post-transplant vitamin D status in lung transplant recipients (LTR), with a specific focus on clinical outcomes and prognosis.

## Materials and Methods

### Study Participants

**FIGURE 1:**
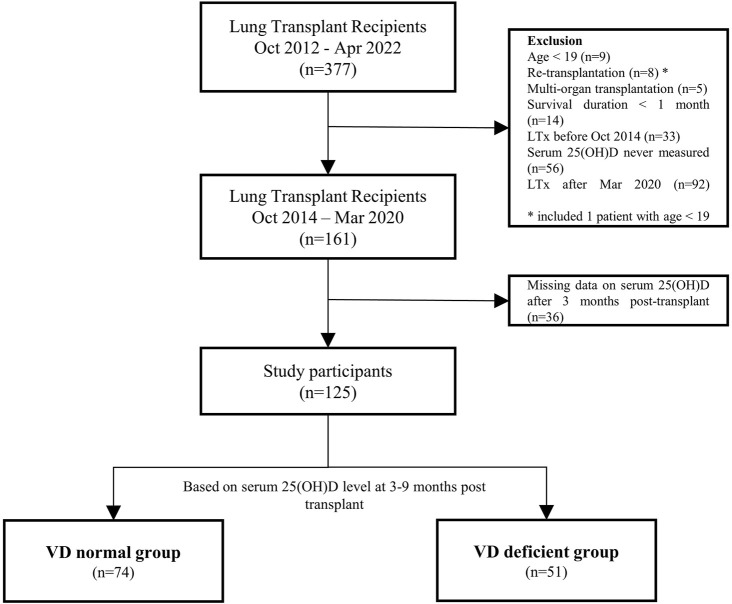
Flowchart of the study participants. LTx, lung transplantation; 25(OH)D, 25-hydroxyvitamin D.

The study included adult patients who underwent lung transplantation at a tertiary hospital in Seoul, South Korea, between October 2014 and March 2020. Exclusions comprised cases of retransplantation, multi-organ transplantation, and patients with a survival duration of less than 1 month. Post-transplant vitamin D status was determined based on levels measured 3–9 months after lung transplantation. Thirty-six transplant recipients missing vitamin D level data at this specific point were excluded from the analysis (Figure 1).

### Determination of Vitamin D Status

Vitamin D status was determined by measuring serum 25(OH)D according to the guidelines, and the measurement was performed using the radioimmunoassay instrument in our institution (Dream Gamma-10, Shin Jin Medics Inc., Goyang, South Korea) [17]. In the case of multiple vitamin D values, post-transplant vitamin D was determined as the average. According to the clinical practice guideline of the American Endocrinology Association, patients with serum 25(OH) < 20 ng/mL were assigned as the vitamin D deficient group, and those above that were classified as the vitamin D normal group [17]. Given the lack of an established reference for optimal vitamin D levels in lung transplant recipients, we stratified the study population into tertiles based on their vitamin D levels to examine sequential trends. The cut-off points for the vitamin D tertiles were 18.2 ng/mL and 24.5 ng/mL, with the groups named VD tertile 1, VD tertile 2, and VD tertile 3 in order of increasing levels (Supplementary Figure S1).

This research protocol was approved by the Institutional Review Board of Severance Hospital (IRB number: 4-2020-0228). The appropriate ethics review boards approved the study design, and informed consent was waived.

### Data Collection

Clinical and demographic data, such as age, sex, preoperative body weight and body mass index (BMI), pre-transplant diagnosis, and comorbidities, were examined from electronic medical records. Transplant waiting time, preoperative intensive care unit (ICU) admission, ventilator care, and extracorporeal membrane oxygenation (ECMO) care were also checked.

Operative findings and postoperative complications that occurred within 1 month after lung transplantation were also investigated. The diagnosis of acute rejection was determined based on the International Society for Heart and Lung Transplant (ISHLT) standard guidelines [18]. As variables for postoperative complications, we investigated whether complications occurred by major organs after lung transplantation: respiratory complication [pneumonia, primary graft dysfunction (PGD), respiratory failure including re-intubation and tracheostomy], postoperative acute kidney injury (AKI), renal replacement therapy (RRT) use, bacteremia, infection (bacterial, viral or fungal), neurologic complication, cardiovascular complication, and gastrointestinal complication were examined. PGD was graded according to the International Society of Heart and Lung Transplant (ISHLT) Working Group criteria. The proposed standardized definition of PGD was based on diffuse pulmonary edema in an allograft on a chest radiograph and a PaO_2_/FiO_2_ (P/F) ratio [19]. Based on the kidney disease: improving global outcomes (KDIGO) guideline, AKI is defined as any of the following: an increase in serum creatinine by ≥ 0.3 mg/dL (≥26.5 μmol/L) within 48 h or an increase in serum creatinine to ≥1.5 times the baseline value, which is known or presumed to have occurred within the prior 7 days; or urine volume <0.5 mL/kg/h for 6 h) [20]. Neurologic, cardiovascular, gastrointestinal, and wound-related complications were defined as cases with appropriate intervention after discussion with the lung transplant team and in collaboration with relevant specialists.

We examined vitamin D supplementation (Cholecalciferol or Calcitriol) pre- and post-transplant, including cumulative dosages. The estimated daily vitamin D supplementation was calculated by dividing the total cumulative supplementation by the follow-up period post-transplantation. To assess prognosis based on vitamin D supplementation, subjects were divided into tertiles of cumulative supplementation. The cut-off points were 864,666.7 IU and 2,731,666.7 IU, labeled VD supplement tertiles 1, 2, and 3 in ascending order. Post-transplant tests were also reviewed, including serum C-reactive protein, pulmonary function tests (PFTs), and 6-minute walk tests (6MWT) measured 3–9 months after lung transplantation. According to relevant guidelines, PFT and 6MWT were performed if the patient’s condition was not limited [21, 22]. Among the study participants, LTRs with a survival period of 1 year or more were investigated for the development of bronchiolitis obliterans syndrome (BOS) based on the ISHLT diagnostic criteria published in 2019 [23].

Colonization by *Pseudomonas* and *Aspergillus* was determined based on the presence of these microorganisms in bronchial washing and bronchoalveolar lavage cultures. The cumulative incidence of post-transplant pneumonia episodes was established by identifying cases that fulfilled both criteria: 1) pneumonia detection on Chest computed tomography (CT) scan and 2) intravenous antibiotic administration within 1 week before and after the pneumonia-detected CT scan date.

All LTRs were initially given a triple immunosuppressive therapy that included a calcineurin inhibitor, an antimetabolite or purine synthesis inhibitor, and corticosteroids. Follow-up duration was defined as from the date of lung transplantation to the date of death or the last follow-up. The end date of the survival analysis was 1 September 2021.

### Statistical Analysis

Continuous variables were summarized using means or medians, while categorical variables were represented by counts and percentages. The Student’s t-test or Mann-Whitney test was employed for continuous variables, and the Chi-squared test or Fisher’s exact test for categorical variables was used to compare the two groups. The survival was estimated using the Kaplan-Meier method, and the significance of the difference was assessed using a log-rank test. Univariable and multivariable analysis of overall survival was conducted using the Cox proportional hazard model to identify predictors of overall survival. In the multivariate logistic regression model, continuous variables, including age, estimated blood loss, operation time, total hospitalization, 6MWT distance, and FEV_1_, were categorized by their median values and incorporated into the analysis. Logistic regression analysis was conducted to determine if vitamin D deficiency significantly contributed to the development of pneumonia post-transplantation. P-values less than 0.05 were considered statistically significant. All statistical analyses were performed using R, version 4.1.1 (R Foundation for Statistical Computing).

## Results

Among the 125 LTRs, there were 74 patients (59.2%) in the post-transplant vitamin D normal group (VD normal group) and 51 patients (40.8%) in the vitamin D deficient group (VD deficient group). The VD deficient group exhibited an older average age, a higher rate of male sex, and a higher prevalence of pre-transplant cardiovascular disease compared to the VD normal group. Operative findings revealed a higher proportion of recipients with pleural adhesion in the VD deficient group compared to the VD normal group, along with an increased estimated blood loss during lung transplantation. There was no statistically significant difference in the incidence of postoperative complications between the two groups. Total hospitalization periods for lung transplantation were more extended in the VD deficient group than in the VD normal group (Table 1).

**TABLE 1:** Basic characteristics of lung transplant recipients according to vitamin D status.

	VD normal group	VD deficient group	*p*-value
	(N = 74)	(N = 51)	
Age	52.6 ± 12.2	57.6 ± 11.4	0.021
Male sex, n (%)	42 (56.8%)	43 (84.3%)	0.002
Body weight (kg)	57.0 ± 11.2	59.4 ± 10.5	0.234
BMI (kg/m^2^)	21.2 ± 3.8	21.4 ± 3.5	0.697
Pre-transplant diagnosis, n (%)			0.443
COPD and emphysema	5 (6.8%)	4 (7.8%)	
ILD	52 (70.3%)	40 (78.4%)	
Bronchiectasis	9 (12.2%)	2 (3.9%)	
Others	8 (10.8%)	5 (9.8%)	
Comorbidities, n (%)			
DM	10 (14.1%)	13 (26.0%)	0.159
HTN	8 (11.3%)	8 (16.0%)	0.628
CV	10 (14.1%)	20 (40.0%)	0.002
CKD	7 (9.9%)	6 (12.0%)	0.939
Tuberculosis	25 (34.7%)	14 (28.0%)	0.558
Transplant waiting time (days)	121.0 [41.0; 231.0]	103.0 [44.5; 249.5]	0.890
Preoperative status, n (%)			
Preop ICU admission	29 (39.2%)	23 (45.1%)	0.635
Preop ventilator care	26 (35.1%)	23 (45.1%)	0.350
Preop ECMO care	22 (29.7%)	20 (39.2%)	0.362
Operative findings, n (%)			
Intraoperative ECMO weaning	43 (65.2%)	36 (70.6%)	0.672
Transplantation Type, Double	66 (95.7%)	50 (100.0%)	0.368
Size mismatch, Bronchus, or PA	37 (56.1%)	29 (59.2%)	0.885
Status of pleura, Adhesion	39 (55.7%)	37 (74.0%)	0.063
Estimated blood loss (mL)	1800.0 [1050.0; 3000.0]	2300.0 [1600.0; 3600.0]	0.036
ECMO time (min)	300.0 [280.0; 360.0]	300.0 [248.0; 390.0]	0.825
Operation time (min)	380.9 ± 79.6	407.2 ± 71.5	0.090
Anesthesia time (min)	479.0 ± 84.8	496.8 ± 70.9	0.280
Postop complications, n (%)			
Acute rejection	2 (2.7%)	0 (0.0%)	0.647
Respiratory^a^	30 (42.9%)	28 (54.9%)	0.260
BPF	2 (2.7%)	3 (5.9%)	0.669
Pneumothorax, pleural effusion	9 (12.3%)	7 (13.7%)	1.000
Bronchial stenosis	7 (9.5%)	5 (9.8%)	1.000
PA stenosis	3 (4.1%)	3 (5.9%)	0.965
Postop AKI	4 (5.6%)	8 (15.7%)	0.126
Postop RRT use	5 (6.8%)	4 (7.8%)	1.000
Bacteremia	1 (1.4%)	3 (5.9%)	0.393
Infection	5 (7.0%)	7 (13.7%)	0.360
Neurologic	2 (2.8%)	2 (3.9%)	1.000
Cardiovascular	2 (2.9%)	3 (5.9%)	0.717
Gastrointestinal	8 (11.1%)	7 (13.7%)	0.875
Postop ICU stay (days)	7.0 [5.0; 13.0]	7.0 [4.5; 13.0]	0.670
Total hospitalization (days)	53.5 [31.5; 91.0]	86.0 [40.0; 136.0]	0.011

Values are displayed as median (interquartile range), n (%), or mean ± standard error of the mean where appropriate. BMI, body mass index; COPD, chronic obstructive pulmonary disease; ILD, interstitial lung disease; DM, diabetes mellitus; HTN, hypertension; CV, cardiovascular; CKD, chronic kidney disease; ICU, intensive care unit; ECMO, extracorporeal membrane oxygenation; BPF, bronchopleural fistula; PA, pulmonary artery; AKI, acute kidney injury; RRT, renal replacement therapy.

^a^ Respiratory complications: pneumonia, primary graft dysfunction (PGD), respiratory failure including re-intubation and tracheostomy.

The VD normal group had higher post-transplant vitamin D supplementation rates and a greater cumulative dose of vitamin D supplementation. Post-transplant, the VD deficient group showed significantly higher CRP levels and a shorter 6MWT distance than the VD normal group (Table 2).

**TABLE 2:** Vitamin D measurements, supplementation and post-transplant test results according to vitamin D status.

	VD normal group	VD deficient group	*p*-value
	(N = 74)	(N = 51)	
Post-transplant 25(OH)D (ng/mL)	26.5 ± 5.1	14.7 ± 3.5	<0.001
Post-transplant 25(OH)D (ng/mL)	25.5 [22.3; 29.3]	15.4 [12.1; 17.8]	<0.001
Number of 25(OH)D measurements	2.7 ± 1.1	2.3 ± 0.8	0.023
Delta 25(OH)D^a^	7.3 ± 7.1	0.5 ± 6.9	<0.001
Delta 25(OH)D^a^	7.6 [2.6; 11.8]	0.4 [−3.4; 6.4]	0.001
Preop VD supplementation^b^, n (%)	67 (90.5%)	40 (78.4%)	0.102
Preop VD cumulative dose (IU)	437,800 [181,000; 688,600]	183,000 [94,000; 458,400]	0.008
Postop VD supplementation^b^, n (%)	73 (98.6%)	44 (86.3%)	0.016
Postop VD cumulative dose (IU)	2,713,200 [975,000; 3,766,000]	760,800 [212,500; 1,854,500]	<0.001
Post-transplant tests			
CRP (mg/L)	7.9 ± 14.3	22.5 ± 33.2	0.004
CRP (mg/L)	1.9 [0.7; 7.8]	6.7 [1.4; 33.8]	0.003
FEV1, predicted %	69.9 ± 20.0	68.4 ± 15.9	0.690
FEV1, liter	2.0 ± 0.7	2.0 ± 0.5	0.862
FVC, predicted %	62.9 ± 16.0	59.2 ± 16.0	0.262
FVC, liter	2.4 ± 0.8	2.4 ± 0.7	0.953
DLCO, predicted %	66.0 ± 21.4	63.0 ± 18.1	0.526
6MWT distance (m)	384.5 ± 129.0	320.0 ± 149.0	0.027

Values are displayed as median (interquartile range), n (%), or mean ± standard error of the mean where appropriate. CRP, C-reactive protein; FEV1, forced expiratory volume in 1 s; FVC, forced vital capacity; DLCO, diffusing capacity of the lungs for carbon monoxide; 6MWT, 6-minute walking test.

^a^ Delta 25(OH)D = post-transplant 25(OH)D - pre-transplant 25(OH)D.

^b^ VD supplementation: Cholecalciferol or Calcitriol.

The average follow-up period for the study participants was 35 months. During the follow-up period, the two groups had no statistically significant differences in the incidence of BOS, *Pseudomonas*, and *Aspergillus* colonization. However, the VD deficient group exhibited significantly higher rates of post-transplant pneumonia and a greater cumulative number of post-transplant pneumonia. The VD deficient group experienced a higher overall mortality rate during the follow-up duration compared to the VD normal group (20.3% vs. 51.0%, *p =0.001*), with infection identified as the primary cause of death in both groups (Table 3).

**TABLE 3:** Overall mortality rate and incidence of infection/rejection of lung transplant recipients according to vitamin D status.

	VD normal group	VD deficient group	*p*-value
	(N = 74)	(N = 51)	
Follow-up duration, months	46.1 ± 26.0	33.4 ± 22.9	0.006
Follow-up duration, months	41.5 [28.0; 68.0]	32.0 [13.0; 42.5]	0.008
BOS^a^, n (%)	18 (26.1%)	8 (17.0%)	0.356
*Pseudomonas* colonization, n (%)	20 (27.0%)	14 (27.5%)	1.000
Aspergillus colonization, n (%)	13 (17.6%)	13 (25.5%)	0.396
Post-transplant pneumonia, n (%)	31 (41.9%)	37 (72.5%)	0.001
Cumulative episodes of post-transplant pneumonia	0.0 [0.0; 2.0]	1.0 [0.0; 3.5]	0.001
Cumulative episodes of post-transplant pneumonia	1.1 ± 1.9	2.1 ± 2.6	0.014
Overall mortality, n (%)	15 (20.3%)	26 (51.0%)	0.001
Cause of death, n (%)			0.750
Sepsis/Infection	10 (66.7%)	17 (65.4%)	
Neurologic	0 (0.0%)	1 (3.8%)	
Hematologic	1 (6.7%)	2 (7.7%)	
Cardiac	1 (6.7%)	1 (3.8%)	
GI	1 (6.7%)	0 (0.0%)	
Miscellaneous	2 (13.3%)	5 (19.2%)	

Values are displayed as median (interquartile range), n (%), or mean ± standard error of the mean where appropriate. BOS, bronchiolitis obliterans syndrome; GI, gastrointestinal.

^a^ Investigated among patients with a survival period of more than 1 year.

**FIGURE 2:**
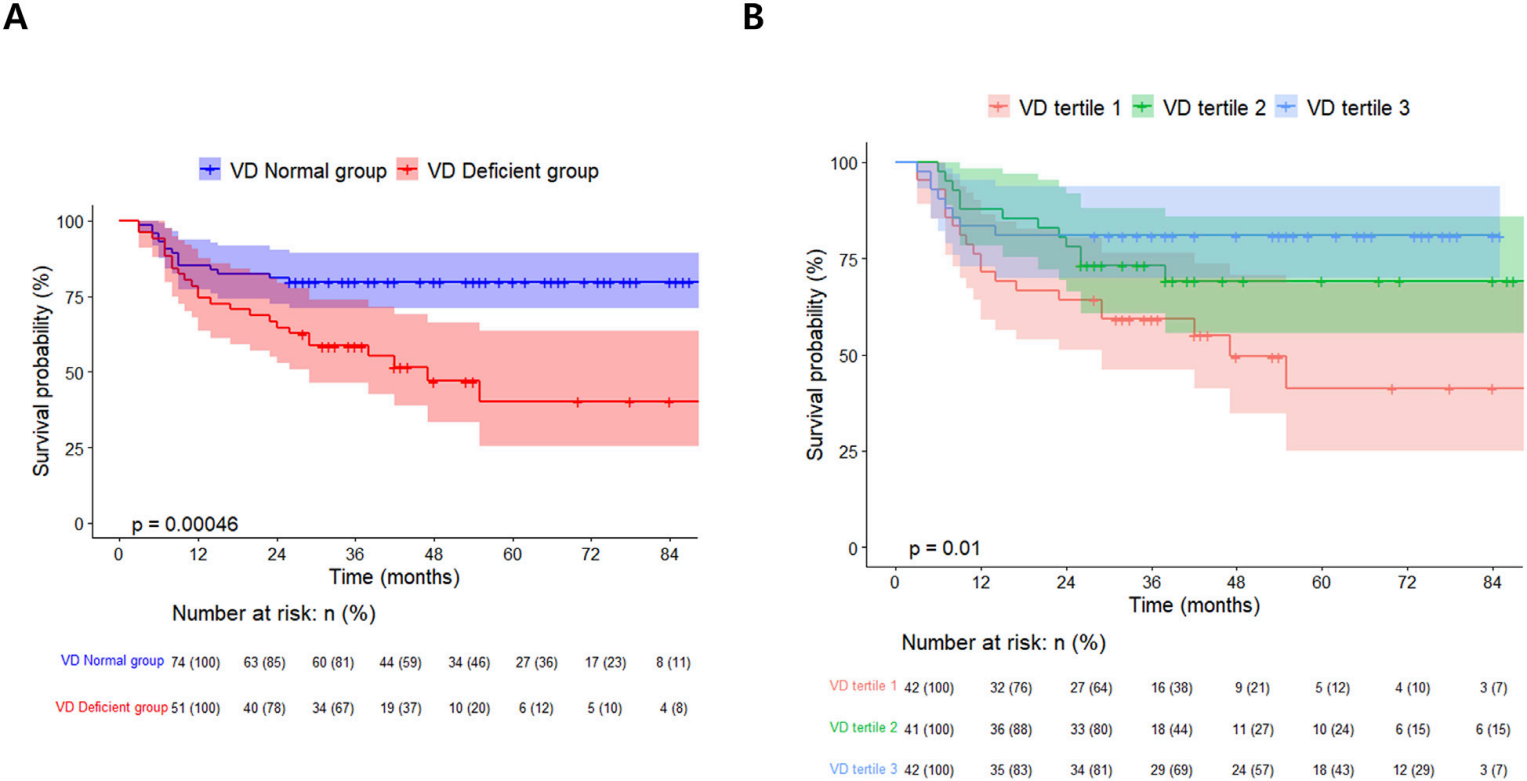
Kaplan-Meier survival curves for lung transplant recipients: **(A)** by post-transplant vitamin D status and **(B)** stratified by Vitamin D tertiles.

In the survival analysis, the VD deficient group showed a lower survival rate than the VD normal group (log-rank test, *p < 0.001*, Figure 2A). The univariate and multivariate Cox proportional hazard analyses were conducted, including post-transplant vitamin D status and covariates that showed statistically significant differences in the two groups. Variables with significant missing values (e.g., post-transplant FEV_1_, post-transplant 6MWT; 20 or more missing) or notable correlations (e.g., total hospitalization, estimated blood loss) were selected for inclusion in the Cox proportional hazards regression model. Following the multivariate analysis, post-transplant VD deficiency [adjusted hazard ratio (aHR) 2.22, 95% confidential interval (CI) 1.05–4.69, *p = 0.036*] and higher CRP level (aHR 9.38, 95% CI 3.61–24.4, *p < 0.001*) emerged as factors significantly related with the prognosis of lung transplant recipients (Table 4).

**TABLE 4:** Cox proportional hazard analysis for lung transplant recipients’ survival.

Variables	Univariable			Multivariable		
	HR	95% CI	*p*-value	aHR	95% CI	*p*-value
Age ≥ 58 (vs. < 58)	2.5	1.29–4.85	0.007	2.33	1.13–4.82	0.022
Female (vs. male)	1.09	0.57–2.08	0.793	1.96	0.89–4.30	0.094
BMI (kg/m^2^)	1.01	0.93–1.10	0.784			
Cardiovascular disease: Presence (vs. Absence)	1.09	0.53–2.23	0.823			
Status of pleura: Adhesion (vs. Normal)	1.19	0.62–2.29	0.597			
Estimated Blood loss (mL) ≥ 2,000 (vs. < 2,000)	3.58	1.78–7.18	<0.001	1	1.00–1.00	0.002
Operation time (min) ≥ 377 (vs. < 377)	1.97	0.93–4.16	0.077			
Total Hospitalization (days) ≥ 61 (vs. < 61)	4.05	1.95–8.38	<0.001			
Post-transplant VD: Deficient (vs. Normal)	2.96	1.56–5.62	0.001	2.22	1.05–4.69	0.036
CRP (mg/L) > 3.1 (vs. < 3.1)	1.06	1.04–1.07	<0.001	9.38	3.61–24.4	<0.001
6MWT distance (m) < 375 (vs. ≥ 375)	3.77	1.35–10.53	0.011			
FEV1, predicted (%) < 70 (vs. ≥ 70)	3.21	1.26–8.22	0.015			
Post-transplant pneumonia: Presence (vs. Absence)	1.9	0.98–3.67	0.057	0.64	0.29–1.42	0.273
Cumulative episodes of post-transplant pneumonia	1.09	0.98–1.20	0.108			

HR, hazard ratio; aHR, adjusted hazard ratio; CI, confidence interval; BMI, body mass index; CRP, C-reactive protein; 6MWT, 6-minute walk test; FEV_1_, forced expiratory volume in 1 s.

### Comparisons of the Vitamin D Tertiles

The results of the comparison divided into vitamin D level tertiles also showed that the lower the vitamin D level, the higher the age, the higher the male ratio, and the higher the rate of cardiovascular disease. Otherwise, there were no significant differences between vitamin D tertiles in the remaining baseline characteristics (Supplementary Table S1). The differences in vitamin D supplementation and post-transplant test results among the vitamin D tertiles mirrored those observed in the VD deficient/normal group. The estimated daily vitamin D supplementation doses for tertiles 1, 2, and 3 were approximately 871 IU, 1685 IU, and 1884 IU, respectively (Supplementary Table S2).

**FIGURE 3:**
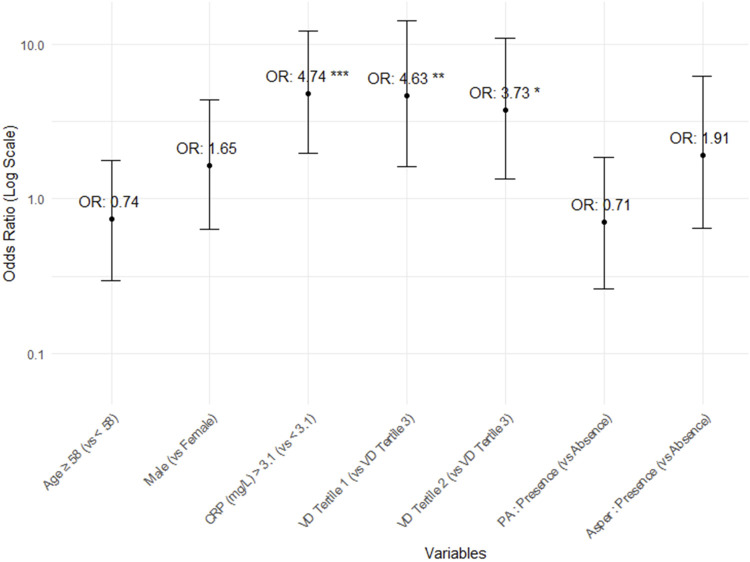
Odds ratios and 95% confidence intervals for the occurrence of post-transplant pneumonia.

In the vitamin D tertiles, lower vitamin D levels were linked to a higher incidence of post-transplant pneumonia over a shorter follow-up period. Additionally, a significant difference in the overall mortality rate was observed, demonstrating a sequential trend related to vitamin D levels [VD tertile 1: 21/42 (50.0%), VD tertile 2: 12/41 (29.3%), VD tertile 3: 8/42 (19.0%); *p = 0.009*, Supplementary Table S3]. Among vitamin D tertiles 1, 2, and 3, a poorer survival curve was observed at lower vitamin D levels (log-rank test, *p = 0.01*, Figure 2B). Multivariate Cox proportional hazards analysis using vitamin D tertiles indicated that VD tertile 1 demonstrated a marginally significant hazard ratio in comparison to VD tertile 3 (aHR 2.45, 95% CI 0.92–6.54, *p = 0.074*, Supplementary Table S4). Logistic regression analysis of post-transplant pneumonia occurrence indicated that lower post-transplant vitamin D levels and higher post-transplant CRP levels were significant covariates (Supplementary Table S5; Figure 3).

### Comparisons of the Vitamin D Supplement Tertiles

**FIGURE 4:**
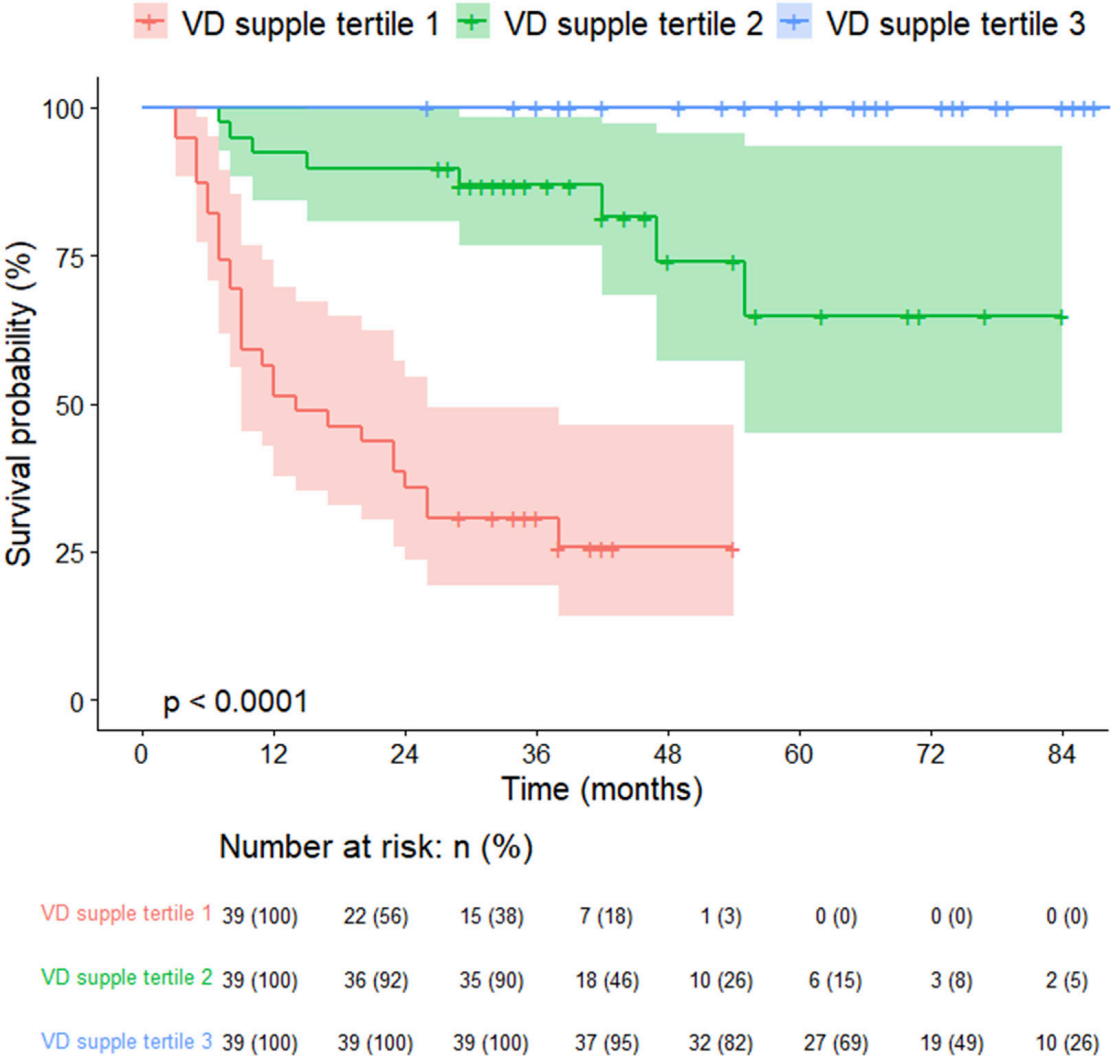
Kaplan-Meier survival curves for lung transplant recipients by vitamin D supplementation tertiles.

The 125 lung transplant recipients were categorized into three tertiles of cumulative vitamin D supplementation after lung transplantation (VD supplement tertile 1, VD supplement tertile 2, and VD supplement tertile 3), with higher cumulative doses resulting in greater vitamin D levels. These tertiles’ estimated daily supplementation doses were 747 IU, 1735 IU, and 1934 IU, respectively (Table 5). The group receiving higher vitamin D supplementation demonstrated improved post-transplant lung function, a reduced incidence of pneumonia, and lower mortality rates. However, the frequency of BOS exhibited an opposite trend (Table 5). The survival curves of lung transplant recipients (LTRs) who received less vitamin D supplementation were inferior to those of LTRs who received higher doses of vitamin D (log-rank test, *p < 0.001,* Figure 4).

**TABLE 5:** Clinical outcomes of lung transplant recipients by vitamin D supplementation tertiles.

	VD supplement tertile 1	VD supplement tertile 2	VD supplement tertile 3	*p*-value
	(N = 39)	(N = 39)	(N = 39)	
Post-transplant 25(OH)D (ng/mL)	18.9 [15.8; 22.6]	21.3 [17.1; 26.6]	25.5 [21.7; 29.0]	<0.001
Number of 25(OH)D measurements	2.3 ± 1.0	2.6 ± 1.0	2.8 ± 1.0	0.101
Postop VD cumulative dose (IU)	269,000 [181,500; 639,000]	1,820,000 [1,394,000; 2,369,200]	3,821,000 [3,179,500; 4,599,600]	<0.001
Estimated daily VD supplement dose (IU)	747.2 [251.6; 982.3]	1,734.5 [1,160.5; 2,120.9]	1,933.7 [1,565.1; 2,643.2]	<0.001
Post-transplant tests				
CRP (mg/L)	14.2 [4.3; 36.2]	2.4 [1.2; 7.3]	1.2 [0.5; 3.8]	<0.001
FEV_1_, predicted %	63.2 ± 19.4	64.5 ± 16.9	76.3 ± 18.1	0.006
FEV_1_, liter	1.8 ± 0.6	1.9 ± 0.7	2.1 ± 0.7	0.119
FVC, predicted %	56.0 ± 18.2	56.4 ± 15.0	67.8 ± 13.2	0.002
FVC, liter	2.2 ± 0.8	2.2 ± 0.8	2.6 ± 0.7	0.075
DLCO, predicted %	61.5 ± 18.0	54.5 ± 20.9	73.0 ± 18.9	0.001
6MWT distance (m)	327.4 ± 162.8	344.9 ± 139.5	386.7 ± 129.6	0.238
Follow up duration, months	14.0 [7.5; 33.0]	35.0 [29.0; 47.5]	68.0 [54.0; 81.5]	<0.001
BOS, n (%)^a^	5 (13.9%)	5 (13.5%)	16 (44.4%)	0.002
*Pseudomonas* colonization, n (%)	12 (30.8%)	10 (25.6%)	11 (28.2%)	0.881
Aspergillus colonization, n (%)	12 (30.8%)	6 (15.4%)	7 (17.9%)	0.207
Post-transplant pneumonia, n (%)	28 (71.8%)	20 (51.3%)	16 (41.0%)	0.021
Cumulative episodes of post-transplant pneumonia	2.0 [0.0; 2.0]	1.0 [0.0; 2.0]	0.0 [0.0; 1.0]	0.040
Cumulative episodes of post-transplant pneumonia	1.8 ± 2.1	1.8 ± 2.8	1.1 ± 2.1	0.352
1-year mortality, n (%)	17 (43.6%)	3 (7.7%)	0 (0.0%)	<0.001
3-year mortality, n (%)	27 (69.2%)	5 (12.8%)	0 (0.0%)	<0.001
Overall mortality, n (%)	28 (71.8%)	8 (20.5%)	1 (2.6%)	<0.001

Cut-off points for Vitamin D supplementation were 864,666.7 IU and 2,731,666.7 IU, creating VD supplement tertiles 1 (≤864,666.7 IU), 2 (864,666.8–2,731,666.7 IU), and 3 (≥2,731,666.8 IU). Estimated daily supplementation doses were 747 IU, 1,735 IU, and 1,934 IU for tertiles 1, 2, and 3, respectively.

Values are displayed as median (interquartile range), n (%), or mean ± standard error of the mean where appropriate. CRP, C-reactive protein; FEV1, forced expiratory volume in 1 s; FVC, forced vital capacity; DLCO, diffusing capacity of the lungs for carbon monoxide; 6MWT, 6-minute walking test; 25(OH)D, 25-hydroxyvitamin D; IU, international unit; BOS, bronchiolitis obliterans syndrome; GI, gastrointestinal.

^a^ Investigated among patients with a survival period of more than 1 year.

## Discussion

In this study, deficiencies in vitamin D levels were observed in a significant number of lung transplant recipients despite high rates of vitamin D supplementation, with differences in frequency of post-transplant pneumonia, overall mortality and survival rates based on post-transplant vitamin D status. Stratifying subjects into vitamin D tertiles revealed a sequential trend in outcomes based on vitamin D levels. Post-transplant vitamin D levels and CRP significantly influenced pneumonia incidence and survival. Additionally, the prognosis varied with the cumulative vitamin D supplementation after transplantation.

Vitamin D is a fat-soluble vitamin absorbed by the body through food (20%) or synthesized in the skin (80%) from 7-dihydrocholesterol after ultraviolet B-ray exposure [24]. Vitamin D from food and skin is hydroxylated in the liver and converted to 25-hydroxyvitamin D [25(OH)D], which has a long half-life and is used to measure and evaluate the vitamin D status of patients. 25(OH)D is metabolized once more in the kidneys to the fully active form of 1,25-dihydroxyvitamin D [1,25(OH)D], which is closely controlled by blood parathyroid hormone and calcium/phosphate levels [25, 26]. Physiologically activated vitamin D mediates various physiological functions by acting on bone, immune cells, and target cells of various organs [27].

Low vitamin D levels have been observed in various disease groups, including end-stage lung diseases and LTRs. Vitamin D deficiency has been reported in 20%–50% of patients with advanced lung disease and in up to two-thirds of patients waiting for lung transplantation [28–31]. In these individuals, inadequate vitamin D levels are associated with lower fat mass, obstructive pulmonary disease, insufficient dietary vitamin D intake, and limited sunlight exposure and have been investigated as predictors of reduced walking distance [31, 32]. Poor health conditions after transplantation, changes in vitamin D metabolism due to glucocorticoid use, and limited sun exposure due to increased risk of skin cancer may further lower vitamin D levels in lung transplant recipients [33, 34].

This study identified post-transplant vitamin D status as an independent variable related to survival. Studies of liver and kidney transplant recipients have also identified post-transplant vitamin D status as an independent factor associated with prognosis [12, 35]. Vitamin D levels after solid organ transplantation appear to reflect the patient’s clinical course, and more needs to be discovered to determine its relationship to prognosis.

In this study, the LTRs with post-transplant vitamin D deficiency had poorer baseline characteristics, including older age, comorbidities such as cardiovascular disease, and surgical findings like pleural adhesion, longer operation times, and extended hospitalization periods. Additionally, they experienced more pneumonia episodes during follow-up.

Lung transplant prognosis is influenced by pre-transplant characteristics and intraoperative factors, with their impact evolving during the post-transplant period [36]. The key prognostic factors include: recipient factors such as age, sex, BMI, pre-transplant diagnosis, ECMO or ventilator use, hospitalization, pulmonary hypertension, and malnutrition; donor factors, including donor age and donor-recipient weight/height mismatch; procedural factors such as ischemic time, severe bleeding, and pleural adhesions; and post-transplant factors, including ECMO requirement, infection, PGD, BOS/chronic lung allograft dysfunction (CLAD), and immunosuppression levels [37]. Previous studies have also shown that prolonged ischemic time, massive bleeding due to pleural adhesions, and other factors are associated with poor prognosis in lung transplant patients [37]. Despite these factors indicating potentially poorer functional status and lower survival rates, low vitamin D status emerged as a significant prognostic factor alongside CRP in multivariate analysis, highlighting its independent impact on outcomes.

In this study, the group with higher vitamin D levels exhibited a greater frequency of BOS, though this was not statistically significant. Additionally, a higher frequency of BOS was observed in those receiving greater cumulative supplementation doses. This trend may be attributed to the longer follow-up period, which could lead to increased BOS diagnoses among patients with higher vitamin D levels and supplementation. Our study had a median follow-up period of 35 months, and according to the ISHLT report, about 65% of transplant recipients did not develop BOS at this time [38]. Considering the complex mechanism and diagnostic process of BOS [23, 39], it seems necessary to figure out the link between vitamin D status and the development of BOS through a sufficiently extended follow-up period.

Similar to the study by Lowery et al., the LTRs with low vitamin D had more episodes of pneumonia after lung transplantation in this study [14]. Considering that the majority of patients who died in this study were due to infection, frequent cases of infection may have contributed to the poor prognosis of the LTRs. Infection is the most common cause of death within the first year after lung transplantation and the second most common cause of death between one and 5 years after transplantation [40].

The association between vitamin D levels and prognosis within 5 years after lung transplantation can primarily be attributed to vitamin D’s protective effects against infections. The activated form of vitamin D, 1,25(OH)D, produced by CYP27B1 (the 25-hydroxyvitamin D 1α-hydroxylase), in various peripheral tissues, initiates signaling pathways that regulate both innate and adaptive immune responses [41]. This signaling enhances the expression of genes crucial for innate immune defense, including those coding for cytokines, chemokines, antimicrobial peptides, and pattern recognition receptors [41]. Additionally, 1,25(OH)D promotes bacterial killing and viral clearance through autophagy, playing a vital role in human defense mechanisms beyond skeletal health [41].

Epidemiological studies and randomized controlled trials have highlighted vitamin D’s protective effects against respiratory infections [42, 43], which may be especially significant for lung transplant recipients immunocompromised due to medications, prolonged hospitalization, and malnutrition. Given that infections are a leading cause of mortality in the years following transplantation [40], vitamin D deficiency could lead to increased infection rates and poorer outcomes in these patients.

This study’s logistic regression analysis demonstrated that low vitamin D levels were associated with higher instances of pneumonia post-transplant. Frequent pneumonia hospitalizations correlate with adverse outcomes, including reduced lung function, diminished quality of life, and increased mortality. Thus, vitamin D deficiency likely exacerbates lung transplant recipients’ already compromised infection defense mechanisms.

Although vitamin D supplementation has not shown overall health benefits in clinical studies for various chronic diseases, it has been reported to result in some extraskeletal benefits, such as reduced infections and increased lung function, in patients with profound vitamin D deficiency [43, 44]. Several clinical trials have been conducted in solid organ transplant recipients to investigate the clinical benefits of correcting vitamin D deficiency [16, 44, 45]. In a clinical trial targeting LTRs, high-dose vitamin D supplementation failed to prove a clinical benefit in chronic lung allograft dysfunction prevalence, overall survival, pulmonary function, acute rejection, and respiratory infections [16]. In the previous trial, the placebo group also received a standard-dose vitamin D supplementation and maintained serum 25(OH)D levels above 30 ng/mL 1 year after lung transplantation, limiting the interpretation of the clinical significance of the much lower vitamin D levels [16]. Considering vitamin D’s impact on the immune system and inflammatory cascade [46, 47], maintaining adequate vitamin D levels after lung transplantation may be necessary in reducing the risk of infection and improving prognosis. Further exploration into how vitamin D deficiency intertwines with infections and prognosis in the intricate immune context of lung transplant recipients is warranted. Additionally, research into optimal vitamin D levels and supplementation dosages in LTRs holds clinical promise.

Most LTRs in this study received vitamin D supplementation, but doses varied widely. Given the lack of clear guidelines on appropriate supplementation doses, we compared prognoses based on these doses. The tertile of LTRs receiving the highest supplementation had an estimated daily intake exceeding the recommended 1,000 IU and achieved serum 25(OH)D levels in the mid-20s ng/mL [48].

A trend of increased pneumonia and poorer prognosis was noted at vitamin D levels below 10–20 ng/mL. Drawing from previous randomized controlled trials [16] and research in other fields [49], further investigation is needed to determine the optimal vitamin D levels for effective infection defense in lung transplant recipients. This research would enable tailored supplementation and management strategies in vitamin D deficiency. Larger studies focusing on the prognosis of lung transplant recipients with vitamin D deficiency could also enhance predictions and outcomes in this population.

This study has several limitations. It examined only 125 lung transplant recipients (LTRs) from a single center, which limits its generalizability and applicability to broader populations. Additionally, the relatively short follow-up period restricts our ability to assess the relationship between low vitamin D levels and chronic rejection. Variability in the timing of vitamin D measurements among patients and the reliance on prescription history rather than actual dosing for vitamin D supplementation further complicate the findings. Despite these limitations, this study highlights the clinical significance of vitamin D deficiency in relation to short-term outcomes after lung transplantation. It also suggests a potential supplementation dose that could serve as a foundation for future large-scale studies to determine optimal vitamin D levels and supplementation strategies.

## Conclusion

This study highlights the significant impact of vitamin D deficiency on clinical outcomes in lung transplant recipients, emphasizing the need for further exploration of its role, optimal levels, and supplementation strategies in this population.

## Data Availability

The raw data supporting the conclusions of this article will be made available by the authors, without undue reservation.

## Supplementary Material

Supplementary Figure 1 Flow diagram of study participants by vitamin D tertiles. Cut-off points for Vitamin D tertiles were 18.2 ng/mL and 24.5 ng/mL, classifying participants into VD tertile 1 (≤18.2 ng/mL), VD tertile 2 (18.3–24.5 ng/mL), and VD tertile 3 (≥24.6 ng/mL).

## Abbreviations

25(OH)D, 25-hydroxyvitamin; 6MWT, six-minute walk test; AKI, acute kidney injury; BOS, bronchiolitis obliterans syndrome; BPF, bronchopleural fistula; ECMO, extracorporeal membrane oxygenation; FEV1, forced expiratory volume in 1 sec; ICU, intensive care unit; LTRs, lung transplant recipients; PFT, pulmonary function test; PGD, primary graft dysfunction; RRT, renal replacement therapy.
