# Supplementary figures and images for: Post-Transplant Vitamin D Deficiency in Lung Transplant Recipients: Impact on Outcomes and Prognosis
Source: Transpl Int. 2024 Oct 25;37:13313. doi: 10.3389/ti.2024.13313 (PMC11543412; doi:10.3389/ti.2024.13313)

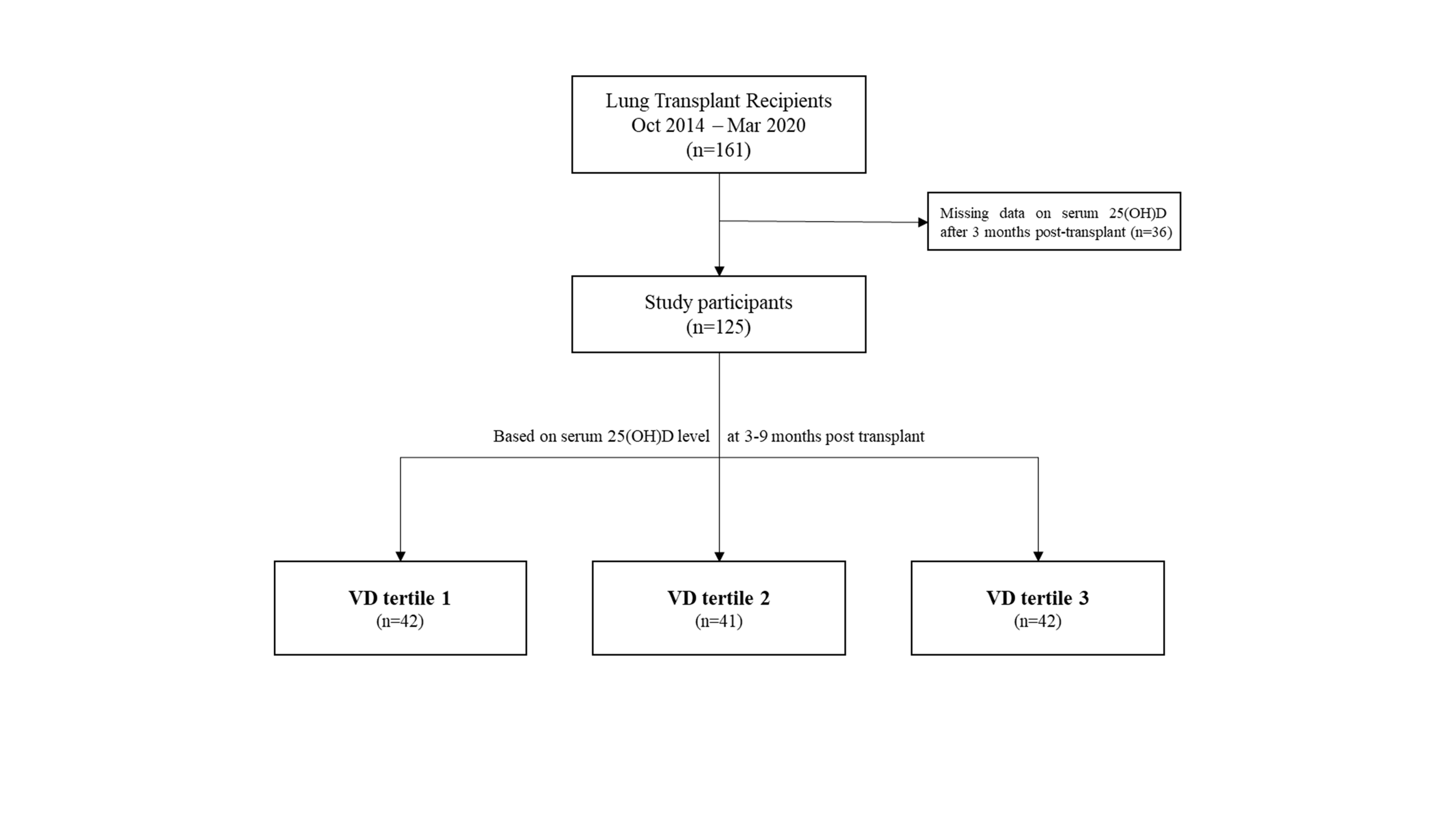

Supplement: Supplementary file 1 [file Image1.JPEG]
