# Supplementary material for: Post-Transplant Vitamin D Deficiency in Lung Transplant Recipients: Impact on Outcomes and Prognosis
Source: Transpl Int. 2024 Oct 25;37:13313. doi: 10.3389/ti.2024.13313 (PMC11543412; doi:10.3389/ti.2024.13313)
Supplement: Supplementary file 2 [file DataSheet1.docx]

**Post-Transplant Vitamin D Deficiency in Lung Transplant Recipients: Impact on Outcomes and Prognosis**

Min Seo Ki, MD^1, 5^, Nam Eun Kim, MD^2^, Ala Woo, MD^1^, Song Yee Kim, MD, PhD^1^, Young Sam Kim, MD, PhD^1^, Ha Eun Kim, MD^3^, Jin Gu Lee, MD, PhD^3^, Hyo Chae Paik, MD, PhD^3, 4^, Moo Suk Park, MD, PhD^1*^

**Supplementary Tables:**

**Supplementary Table 1. Basic characteristics of lung transplant recipients by vitamin D tertiles.**

|  | VD tertile 1 | VD tertile 2 | VD tertile 3 | p-value |
| --- | --- | --- | --- | --- |
|  | (N=42) | (N=41) | (N=42) |  |
| Age | 58.3 ± 10.6 | 54.2 ± 11.7 | 51.5 ± 13.0 | 0.031 |
| Male sex, n (%) | 35 (83.3%) | 26 (63.4%) | 24 (57.1%) | 0.027 |
| Body weight (kg) | 59.7 ± 10.8 | 59.1 ± 11.6 | 55.3 ± 10.1 | 0.141 |
| BMI (kg/m2) | 21.7 ± 3.7 | 21.9 ± 3.5 | 20.3 ± 3.8 | 0.108 |
| Pre-transplant diagnosis, n (%) |  |  |  | 0.253 |
| COPD & emphysema | 3 ( 7.1%) | 3 ( 7.3%) | 3 ( 7.1%) |  |
| ILD | 32 (76.2%) | 34 (82.9%) | 26 (61.9%) |  |
| Bronchiectasis | 2 ( 4.8%) | 3 ( 7.3%) | 6 (14.3%) |  |
| Others | 5 (11.9%) | 1 ( 2.4%) | 7 (16.7%) |  |
| Cormobidities, n (%) |  |  |  |  |
| DM | 11 (26.8%) | 7 (17.5%) | 5 (12.5%) | 0.248 |
| HTN | 6 (14.6%) | 7 (17.5%) | 3 ( 7.5%) | 0.396 |
| CV | 17 (41.5%) | 9 (22.5%) | 4 (10.0%) | 0.004 |
| CKD | 6 (14.6%) | 2 ( 5.0%) | 5 (12.5%) | 0.341 |
| Tuberculosis | 10 (24.4%) | 10 (25.0%) | 19 (46.3%) | 0.053 |
| Transplant waiting time (days) | 107.0 [45.0;254.0] | 94.0 [43.0;301.0] | 122.5 [35.0;208.0] | 0.970 |
| Preoperative status, n (%) |  |  |  |  |
| Preop ICU admission | 18 (42.9%) | 18 (43.9%) | 16 (38.1%) | 0.848 |
| Preop ventilator care | 20 (47.6%) | 15 (36.6%) | 14 (33.3%) | 0.373 |
| Preop ECMO care | 17 (40.5%) | 15 (36.6%) | 10 (23.8%) | 0.240 |
| Operative findings, n (%) |  |  |  |  |
| Intraoperative ECMO weaning | 30 (71.4%) | 21 (56.8%) | 28 (73.7%) | 0.234 |
| Transplantation Type, Double | 41 (100.0%) | 36 (94.7%) | 39 (97.5%) | 0.329 |
| Size mismatch (Bronchus, PA) | 25 (62.5%) | 18 (52.9%) | 23 (56.1%) | 0.694 |
| Status of pleura, Adhesion | 28 (68.3%) | 24 (63.2%) | 24 (58.5%) | 0.657 |
| Estimated blood loss | 2,000 [1,500;3,600] | 2,075 [1,500;3,250] | 1,800 [1,100;2,800] | 0.191 |
| ECMO time | 300.0 [240.5;390.0] | 302.5 [300.0;380.0] | 295.0 [260.0;360.0] | 0.318 |
| Operation time | 400.6 ± 70.9 | 395.5 ± 75.8 | 380.5 ± 84.6 | 0.540 |
| Anesthesia time | 493.0 ± 73.0 | 488.8 ± 81.1 | 478.8 ± 84.8 | 0.763 |
| Postop complications, n (%) |  |  |  |  |
| Acute rejection | 0 ( 0.0%) | 2 ( 4.9%) | 0 ( 0.0%) | 0.125 |
| Operation site bleeding | 7 (16.7%) | 6 (14.6%) | 2 ( 4.8%) | 0.200 |
| Respiratory ^*^ | 26 (61.9%) | 16 (41.0%) | 16 (40.0%) | 0.080 |
| BPF | 3 ( 7.1%) | 0 ( 0.0%) | 2 ( 4.8%) | 0.240 |
| Pneumothorax, pleural effusion | 7 (16.7%) | 5 (12.2%) | 4 ( 9.8%) | 0.635 |
| Bronchial stenosis | 4 ( 9.5%) | 4 ( 9.8%) | 4 ( 9.5%) | 0.999 |
| PA stenosis | 3 ( 7.1%) | 2 ( 4.9%) | 1 ( 2.4%) | 0.594 |
| postop AKI | 7 (16.7%) | 3 ( 7.5%) | 2 ( 5.0%) | 0.173 |
| postop RRT use | 4 ( 9.5%) | 4 ( 9.8%) | 1 ( 2.4%) | 0.333 |
| Bacteremia | 3 ( 7.1%) | 1 ( 2.5%) | 0 ( 0.0%) | 0.182 |
| Infection | 6 (14.3%) | 4 (10.0%) | 2 ( 5.0%) | 0.369 |
| Neurologic | 2 ( 4.8%) | 1 ( 2.5%) | 1 ( 2.4%) | 0.794 |
| Cardiovascular | 3 ( 7.1%) | 2 ( 5.1%) | 0 ( 0.0%) | 0.249 |
| Gastrointestinal | 5 (11.9%) | 6 (15.0%) | 4 ( 9.8%) | 0.769 |
| Postop ICU stay (days) | 8.0 [ 4.0;16.0] | 7.0 [ 5.0;12.0] | 7.5 [ 5.0;12.0] | 0.993 |
| Total hospitalization (days) | 89.5 [45.0;136.0] | 59.0 [32.0;92.5] | 46.0 [29.0;94.0] | 0.020 |

Values are displayed as median (interquartile range), n (%), or mean ± standard error of the mean where appropriate. BMI, body mass index; COPD, chronic obstructive pulmonary disease; ILD, interstitial lung disease; DM, diabetes mellitus; HTN, hypertension; CV, cardiovascular; CKD, chronic kidney disease; ICU, intensive care unit; ECMO, extracorporeal membrane oxygenation; BPF, bronchopleural fistula; PA, pulmonary artery; AKI, acute kidney injury; RRT, renal replacement therapy.

* Respiratory complications: pneumonia, primary graft dysfunction (PGD), respiratory failure including re-intubation and tracheostomy.

**Supplementary Table 2. Vitamin D measurements, supplementation, and post-transplant test results by vitamin D tertiles.**

|  | VD tertile 1 | VD tertile 2 | VD tertile 3 | p-value |
| --- | --- | --- | --- | --- |
|  | (N=42) | (N=41) | (N=42) |  |
| Post-transplant 25(OH)D (ng/ml) | 13.7 [11.4;16.6] | 21.7 [20.4;22.6] | 28.7 [25.9;31.9] | <0.001 |
| Number of 25(OH)D measurements | 2.2 ± 0.8 | 2.8 ± 1.0 | 2.8 ± 1.1 | 0.014 |
| Preop VD supplementation, n (%) | 32 (76.2%) | 40 (97.6%) | 35 (83.3%) | 0.019 |
| Preop VD cumulative dose (IU) | 193,000 [102,500;481,000] | 399,500 [111,500;575,500] | 360,000 [183500;688600] | 0.236 |
| Postop VD supplementation, n (%) | 35 (83.3%) | 41 (100.0%) | 41 (97.6%) | 0.003 |
| Postop VD cumulative dose (IU) | 832,000 [212,500;1,814,400] | 1,770,800 [561,000;3,119,000] | 2,750,400 [1,525,200;3,596,600] | <0.001 |
| Estimated daily VD supplement dose (IU) ^#^ | 871.4 [671.7;1,575.3] | 1,685.9 [896.3;2,082.3] | 1,884.2 [1,335.9;2,532.4] | <0.001 |
| Post-transplant tests |  |  |  |  |
| CRP (mg/L) | 8.1 [ 1.3;35.2] | 3.1 [ 0.9; 7.5] | 2.1 [ 0.6; 8.0] | 0.026 |
| FEV_1_, predicted % | 71.0 ± 16.1 | 65.3 ± 15.8 | 72.0 ± 22.2 | 0.264 |
| FEV_1_, liter | 2.1 ± 0.6 | 1.9 ± 0.6 | 2.0 ± 0.7 | 0.684 |
| FVC, predicted % | 61.8 ± 16.3 | 59.9 ± 15.1 | 62.9 ± 16.9 | 0.730 |
| FVC, liter | 2.4 ± 0.8 | 2.3 ± 0.7 | 2.3 ± 0.8 | 0.800 |
| DLCO, predicted % | 66.5 ± 18.8 | 60.1 ± 16.2 | 68.4 ± 23.9 | 0.241 |
| 6MWT distance (m) | 307.1 ± 139.4 | 379.8 ± 151.0 | 387.0 ± 117.2 | 0.045 |

Values are displayed as median (interquartile range), n (%), or mean ± standard error of the mean where appropriate. CRP, C-reactive protein; FEV1 forced expiratory volume in 1 sec; FVC, forced vital capacity; DLCO, diffusing capacity of the lungs for carbon monoxide; 6MWT, 6-minute walking test. ^#^ VD supplementation: Cholecalciferol or Calcitriol.

**Supplementary Table 3. Overall mortality rate and incidence of infection/rejection in lung transplant recipients by vitamin D tertiles.**

|  | VD tertile 1 | VD tertile 2 | VD tertile 3 | p-value |
| --- | --- | --- | --- | --- |
|  | (N=42) | (N=41) | (N=42) |  |
| Follow up duration, months | 32.0 [12.0;44.0] | 34.0 [26.0;49.0] | 54.5 [32.0;73.0] | 0.024 |
| BOS, n (%) ^*^ | 8 (21.1%) | 5 (12.5%) | 13 (34.2%) | 0.069 |
| Pseudomonas colonization, n (%) | 12 (28.6%) | 10 (24.4%) | 12 (28.6%) | 0.885 |
| Aspergillus colonization, n (%) | 10 (23.8%) | 12 (29.3%) | 4 ( 9.5%) | 0.072 |
| Post-transplant pneumonia, n (%) | 30 (71.4%) | 26 (63.4%) | 12 (28.6%) | <0.001 |
| Cumulative episodes of post-transplant pneumonia | 1.0 [ 0.0; 2.0] | 1.0 [ 0.0; 2.0] | 0.0 [ 0.0; 1.0] | <0.001 |
| Cumulative episodes of post-transplant pneumonia | 2.1 ± 2.8 | 1.6 ± 1.9 | 0.8 ± 1.9 | 0.021 |
| 1-year mortality, n (%) | 10 (23.8%) | 5 (12.2%) | 7 (16.7%) | 0.374 |
| 3-year mortality, n (%) | 17 (40.5%) | 11 (26.8%) | 8 (19.0%) | 0.09 |
| Overall mortality, n (%) | 21 (50.0%) | 12 (29.3%) | 8 (19.0%) | 0.009 |
| Cause of death, n (%) |  |  |  | 0.699 |
| Sepsis/Infection | 14 (66.7%) | 8 (66.7%) | 5 (62.5%) |  |
| Neurologic | 1 ( 4.8%) | 0 ( 0.0%) | 0 ( 0.0%) |  |
| Hematologic | 2 ( 9.5%) | 1 ( 8.3%) | 0 ( 0.0%) |  |
| Cardiac | 0 ( 0.0%) | 1 ( 8.3%) | 1 (12.5%) |  |
| GI | 0 ( 0.0%) | 1 ( 8.3%) | 0 ( 0.0%) |  |
| Miscellaneous | 4 (19.0%) | 1 ( 8.3%) | 2 (25.0%) |  |

Values are displayed as median (interquartile range), n (%), or mean±standard error of the mean where appropriate. BOS, bronchiolitis obliterans syndrome; GI, gastrointestinal.

* Investigated among patients with a survival period of more than one year.

**Supplementary Table 4. Cox proportional hazard analysis for lung transplant recipients’ survival with respect to vitamin D tertiles.**

|  | Univariable | | | Multivariable | | |
| --- | --- | --- | --- | --- | --- | --- |
| Variables | HR | 95%CI | p-value | HR | 95%CI | p-value |
| Age ≥ 58 (vs < 58) | 2.50 | 1.29-4.85 | 0.007 | 2.34 | 1.13-4.86 | 0.022 |
| Female (vs male) | 1.09 | 0.57-2.08 | 0.793 | 1.73 | 0.82-3.64 | 0.147 |
| BMI (kg/m2) | 1.01 | 0.93-1.10 | 0.784 |  |  |  |
| Cardiovascular disease: Presence (vs Absence) | 1.09 | 0.53-2.23 | 0.823 |  |  |  |
| Status of pleura: Adhesion (vs Normal) | 1.19 | 0.62-2.29 | 0.597 |  |  |  |
| Estimated Blood loss (ml) ≥ 2000 (vs < 2000) | 3.58 | 1.78-7.18 | <0.001 | 1.00 | 1.00-1.00 | 0.006 |
| Operation time (min) ≥ 377 (vs < 377) | 1.97 | 0.93-4.16 | 0.077 |  |  |  |
| Total Hospitalization (days) ≥ 61 (vs < 61) | 4.05 | 1.95-8.38 | <0.001 |  |  |  |
| Post-transplant VD: Tertile 1 (vs Tertile 3) | 3.07 | 1.35-6.97 | 0.007 | 2.45 | 0.92-6.54 | 0.074 |
| Post-transplant VD: Tertile 2 (vs Tertile 3) | 1.54 | 0.62-3.79 | 0.352 | 1.42 | 0.52-3.91 | 0.496 |
| CRP (mg/L) > 3.1 (vs < 3.1) | 9.65 | 4.05-23.03 | <0.001 | 10.24 | 3.87-27.1 | <0.001 |
| 6MWT distance (m) < 375 (vs ≥ 375) | 3.77 | 1.35-10.53 | 0.011 |  |  |  |
| FEV1, predicted (%) < 70 (vs ≥ 70) | 3.21 | 1.26-8.22 | 0.015 |  |  |  |
| Post-transplant pneumonia: Presence (vs Absence) | 1.90 | 0.98-3.67 | 0.057 | 0.59 | 0.25-1.39 | 0.228 |
| Cumulative episodes of post-transplant pneumonia | 1.09 | 0.98-1.20 | 0.108 |  |  |  |

HR, hazard ratio; aHR, adjusted hazard ratio; CI, confidence interval; BMI, body mass index; CRP, C-reactive protein; 6MWT, 6-minute walk test; FEV_1_, forced expiratory volume in 1 sec.

**Supplementary Table 5. Univariable and multivariable logistic regression for post-transplant pneumonia.**

|  | Univariable | | | Multivariable | | |
| --- | --- | --- | --- | --- | --- | --- |
| Variables | OR | 95%CI | p-value | OR | 95%CI | p-value |
| Age ≥ 58 (vs < 58) | 1.37 | 0.67-2.83 | 0.392 | 0.74 | 0.30-1.78 | 0.511 |
| Male (vs female) | 1.61 | 0.75-3.51 | 0.223 | 1.65 | 0.64-4.36 | 0.304 |
| BMI (kg/m2) | 0.94 | 0.85-1.04 | 0.247 |  |  |  |
| DM : Presence (vs Absence) | 0.73 | 0.29-1.82 | 0.498 |  |  |  |
| Pre_transplant_diagnosis : ILD (vs COPD) | 0.70 | 0.14-3.04 | 0.642 |  |  |  |
| Pre_transplant_diagnosis : Bronchiectasis (vs COPD) | 1.60 | 0.22-12.1 | 0.637 |  |  |  |
| Pre_transplant_diagnosis : Others (vs COPD) | 0.30 | 0.04-1.85 | 0.206 |  |  |  |
| Total Hospitalization (days) ≥ 61 (vs < 61) | 1.15 | 0.56-2.37 | 0.703 |  |  |  |
| Post-transplant VD: Tertile 1 (vs Tertile 3) | 5.64 | 2.23-15.2 | <0.001 | 4.63 | 1.63-14.1 | 0.005 |
| Post-transplant VD: Tertile 2 (vs Tertile 3) | 3.73 | 1.49-9.78 | 0.006 | 3.73 | 1.35-10.9 | 0.013 |
| CRP (mg/L) > 3.1 (vs < 3.1) | 4.16 | 1.96-9.18 | <0.001 | 4.74 | 1.98-12.1 | <0.001 |
| Pseudomonas colonization : Presence (vs Absence) | 1.10 | 0.50-2.47 | 0.813 | 0.71 | 0.26-1.85 | 0.479 |
| Aspergillus colonization : Presence (vs Absence) | 2.89 | 1.10-8.59 | 0.040 | 1.91 | 0.65-6.17 | 0.252 |

HR, hazard ratio; aHR, adjusted hazard ratio; CI, confidence interval; BMI, body mass index; COPD, chronic obstructive pulmonary disease; ILD, interstitial lung disease; DM, diabetes mellitus; CRP, C-reactive protein.

**Supplementary Figure legends:**

**Supplementary Figure 1. Flow diagram of study participants by vitamin D tertiles.** Cut-off points for Vitamin D tertiles were 18.2 ng/mL and 24.5 ng/mL, classifying participants into VD tertile 1 (≤18.2 ng/mL), VD tertile 2 (18.3–24.5 ng/mL), and VD tertile 3 (≥24.6 ng/mL).
